# Predicting postoperative coronal imbalance in Lenke 1/2 adolescent idiopathic scoliosis: A machine learning model with clinical interpretability

**DOI:** 10.1007/s00264-026-06953-6

**Published:** 2026-07-18

**Authors:** Tao Li, Chaofeng Guo, Sha Li

**Affiliations:** 1 Department of Spine Surgery and Orthopaedics, Xiangya Hospital, Central South University, Changsha, Hunan 410008 China; 2 College of Medicine, Nursing and Health Sciences, University of Galway, Galway, H91 TK33 Ireland; 3 National Clinical Research Center for Geriatric Disorders, Xiangya Hospital, Central South University, Changsha, Hunan 410008 China

**Keywords:** Adolescent idiopathic scoliosis, Coronal imbalance, Machine learning, Preoperative planning, Surgical decision-making, SHAP analysis

## Abstract

**Objectives:**

Selective posterior thoracic fusion (sPTF) for Lenke 1/2 adolescent idiopathic scoliosis (AIS) aims to reconcile multi-planar correction with motion preservation. Nevertheless, postoperative coronal imbalance (CIB) frequently compromises these objectives. This study developed an interpretable machine learning architecture to stratify CIB risk and identify key predictors.

**Methods:**

Data from 282 patients were analyzed. Following dual-stage dimensionality reduction (Boruta and LASSO) on 24 candidate predictors, ten machine learning architectures were trained and evaluated using split-sample internal validation. Model efficacy was evaluated via area under the curve (AUC), Brier score, and decision curve analysis, using SHapley Additive exPlanations (SHAP) framework for algorithmic transparency.

**Results:**

The LightGBM model demonstrated favorable performance, achieving peak AUC of 0.885 (training) and 0.824 (internal validation). SHAP identified three key predictors: the spatial relationship between the lowest instrumented vertebra and the last substantially touching vertebra (LIV-LSTV), regional lumbar adaptability (Lumbar Modifier), and skeletal maturity (Risser grade). Lower LIV-LSTV values, Lumbar Modifiers C, and lower Risser grades were associated with a higher predicted risk of CIB.

**Conclusion:**

This framework incorporates information on distal instrumentation selection, lumbar curve morphology, and skeletal maturity. The model may provide a preliminary basis for future preoperative CIB risk estimation and support a more informed assessment of the trade-off between deformity correction and preservation of lumbar motion segments. Independent external validation is required before the model can be used to guide surgical decision-making.

**Supplementary information:**

The online version contains supplementary material available at https://doi.org/10.1007/s00264-026-06953-6.

## Introduction

The surgical management of Adolescent Idiopathic Scoliosis (AIS), particularly Lenke type 1 and 2 curves treated with selective posterior thoracic fusion (sPTF), requires a delicate balance between multi-planar deformity correction and motion preservation (1–4). However, successful regional curve correction may be compromised by postoperative coronal imbalance (CIB) (5, 6), whose reported incidence varies across different AIS curve patterns and postoperative assessment time points (3, 7). Consequently, coronal balance (CB), alongside sagittal alignment, has emerged as the core evaluative framework for modern surgical efficacy (4). Persistent CIB, defined by an absolute value of Coronal Balance Distance (|CBD|) > 20 mm (3, 8, 9), not only compromises aesthetic outcomes but has been relevant to diminished health-related quality of life (HRQoL)

(10–12) and mechanical hardware failure requiring revision.

Accurately forecasting this complication remains highly challenging due to the intricate, non-linear biomechanics inherent to thoracic-dominant curves. While specific risk factors have been identified, such as type C configurations in degenerative scoliosis (13), these predictors rarely generalize to Lenke 1/2 patients. The unique pelvic compensatory mechanisms of thoracic-major curves may limit the ability of traditional linear models to capture potentially relevant non-linear relationships and interactions. Secondly, the sample size is relatively small. There are few reports on the primary causes and risk factors of CIB in patients with Lenke 1/2 type AIS after corrective surgery, and whether postoperative CIB can be predicted remains unclear.

Recently, machine learning (ML) models have shown potential in predicting postoperative complications by capturing complex relationships that may not be fully represented by conventional statistical approaches (14). To address this evidence gap, the present study developed an interpretable ML-based model for predicting postoperative CIB in patients with Lenke 1/2 AIS, utilizing a single-centre retrospective analysis. By integrating preoperative clinical and radiographic parameters, this approach aims to stratify CIB risk and provide a preliminary data-driven framework for further evaluation in preoperative planning.

## Materials and methods

### Study design and patient selection

**Fig. 1:**
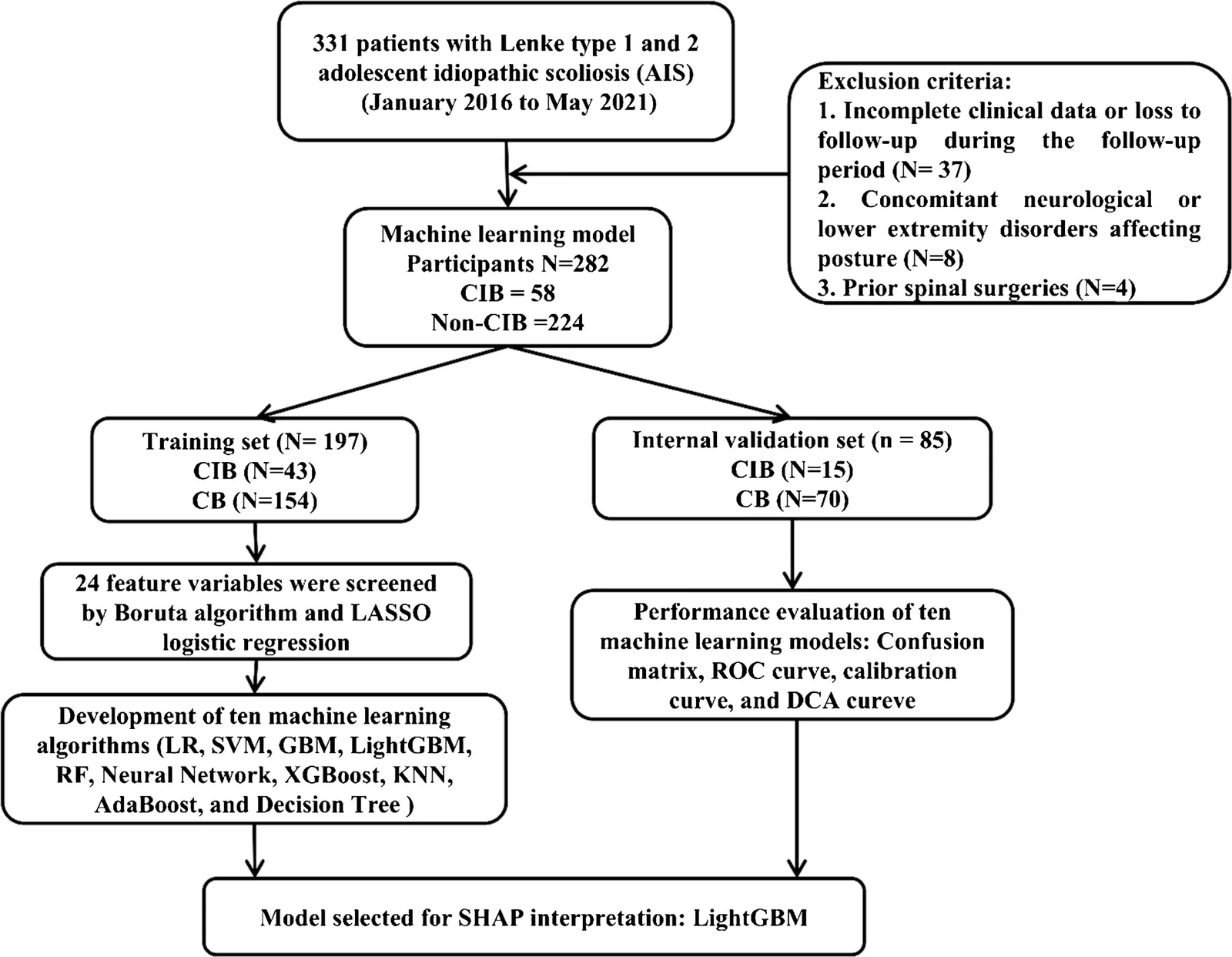
Flowchart of study design and machine learning model development

Following the Ethics Committee approval from the Xiangya Hospital, this single-center retrospective study analyzed 282 patients with Lenke type 1 and 2 AIS who underwent selective posterior thoracic fusion between January 2016 and May 2021. Inclusion criteria were: (1) age ten to 18 years; (2) Lenke 1 or 2 AIS; (3) surgical treatment with sPTF; and (4) ≥ 24 months of postoperative follow-up. Exclusion criteria were: (1) Incomplete clinical data or loss to follow-up during the follow-up period; (2) concomitant neurological or lower extremity disorders affecting posture; and (3) prior spinal surgery. At the last follow-up, the cohort was dichotomized based on |CBD|: a CB group (|CBD|≤ 20 mm) and a CIB group (|CBD|> 20 mm) (8, 9). CBD was defined as the horizontal distance between the C7 plumb line and the central sacral vertical line (CSVL) on standing coronal radiographs. The 20-mm threshold was selected because it has been commonly used in previous AIS studies to define radiographic coronal imbalance (2). The final follow-up assessment was used to evaluate longer-term coronal imbalance rather than immediate post-operative imbalance, which may improve during postoperative adaptation. The follow-up duration did not differ significantly between the CB group (35.87 ± 6.03 months) and the CIB group (35.74 ± 6.00 months) (t = 0.14, *P* = 0.888). The detailed screening process is illustrated in Fig. 1.

### Surgical technique

All patients received individualized sPTF utilizing pedicle screws or transverse process hooks. Upon placement of pre-contoured rods, 3D correction was executed via apical derotation, convex compression, and concave distraction under continuous alignment monitoring. The lowest instrumented vertebra (LIV) was targeted at or one to two segments adjacent to the last substantially touching vertebra (LSTV), and the upper instrumented vertebra (UIV) was anchored one to two levels proximal to the upper end vertebra (UEV). For quantitative encoding, LIV-LSTV was calculated as the vertebral level difference between the LIV and the LSTV. A value of 0 indicated that the LIV was at the same level as the LSTV. Positive values indicated that the LIV was located distal or caudal to the LSTV, whereas negative values indicated that the LIV was located proximal or cranial to the LSTV. For example, LIV-LSTV = 2 indicated that the LIV was two vertebral levels distal to the LSTV.

### Feature engineering and dimensionality reduction

To operationalize the predictive framework, an initial feature space of 24 preoperative candidate predictors was established. This comprehensive dataset integrated demographics (age, sex), skeletal maturity (Risser and triradiate cartilage status), curve morphology (Lenke classification and modifiers), and spatial instrumentation parameters (LIV-LSTV, directionally encoded as described above, UIV-T1). Multi-planar biomechanical characteristics were parameterized via Cobb angles, flexibility indices, and a robust suite of radiographic markers covering shoulder balance (T1 tilt, clavicle angle, radiographic shoulder height), central coronal translation (apical vertebral translation, thoracic trunk shift, LIV-CSVL, baseline Coronal Balance Distance), and sagittal alignment (thoracic kyphosis, lumbar lordosis, sagittal vertical axis). Prior to model development, the cohort was partitioned (training set: internal validation set = 7:3 ratio), and a dual-stage dimensionality reduction pipeline was implemented to isolate high-fidelity predictors. The model was constructed in the training set and then evaluated in the internal validation set. Because both subsets originated from the same institutional cohort, this split-sample validation assessed internal reproducibility rather than external generalizability. The Boruta algorithm was first utilized to determine intrinsic feature significance and mitigate multicollinearity, followed by LASSO regression with tenfold cross-validation (*mlr3verse* and *glmnet*) to optimize the feature subset through penalty-based regularization.

### Algorithmic development and interpretability framework

The refined feature subset served as the input for ten distinct machine learning architectures, encompassing both traditional linear models and advanced non-linear ensembles: Logistic Regression (LR), Support Vector Machine (SVM), Gradient Boosting Machine (GBM), LightGBM, Neural Network, Random Forest (RF), eXtreme Gradient Boosting (XGBoost), K-Nearest Neighbor (KNN), AdaBoost, and Decision Tree. The LR model, based on the same three retained predictors as LightGBM, was included as the conventional reference model to assess whether LightGBM provided incremental predictive value beyond a traditional statistical approach. To rigorously prevent data leakage and ensure optimal generalization, hyperparameter tuning for the non-linear configurations was executed via an exhaustive tenfold cross-validated grid search strictly within the training set. Given the inherent class imbalance between the CIB and CB sets, model efficacy was quantified through a comprehensive and threshold-independent evaluative matrix. Discriminative capacity was primarily assessed using the Area Under the Receiver Operating Characteristic Curve (AUC) and the F1-score. To translate predictive accuracy into surgical reliability, calibration plots and the Brier score were utilized to evaluate the concordance between predicted probabilities and observed CIB frequencies. Additionally, Decision Curve Analysis (DCA) was applied to quantify the standardized net clinical benefit across a continuum of risk thresholds, an essential metric for practical surgical decision-making. Finally, the SHapley Additive exPlanations (SHAP) framework was embedded to resolve the "black-box" nature of advanced ensembles. This game-theory-based approach was utilized to quantify both global predictor importance and patient-specific risk contributions, thereby providing transparent and interpretable evidence for the model's decision-making process (15).

### Statistical analysis

All statistical analyses were conducted using R software (version 4.4.2). Continuous variables were assessed for normality and presented as mean ± standard deviation (SD) or median (Q₁, Q₃), as appropriate. Categorical data were expressed as counts and percentages. To compare baseline characteristics between the CB and CIB groups, univariate analyses (Chi-square tests, independent-sample t-tests, and Mann–Whitney U tests) were employed. Statistical significance was predefined at a P value < 0.05.

## Results

### Patient characteristics and univariate analysis

The study cohort comprised 282 AIS patients, stratified into training (*n *= 197) and internal validation (*n* = 85) sets. No statistically significant differences in the recorded baseline characteristics were observed between the training and internal validation sets (all *P* > 0.05; Table 1). Overall, postoperative CIB occurred in 58 patients. The training cohort included 43 CIB and 154 CB cases, whereas the internal validation cohort included 15 CIB and 70 CB cases. Specifically, patients developing postoperative CIB exhibited significantly lower LC flexibility and fewer LIV-LSTV segments. Furthermore, the CIB group presented with distinct skeletal maturity evidenced by differing Risser grade distributions (*P* = 0.014), and a markedly higher prevalence of advanced Lumbar Modifiers (types B and C) compared to the CB set (*P* < 0.001) (Table 2).

**Table 1:** Characteristics of the Study Cohort Stratified by Training and Internal Validation Sets

Variables	Total (*n *= 282)	Valid (*n *= 85)	Train (*n *= 197)	Statistic	*P*
Age (years), Mean ± SD	14.34 ± 1.84	14.42 ± 1.88	14.30 ± 1.82	t = 0.50	0.619
LIV-LSTV (segments), Mean ± SD	0.41 ± 1.14	0.58 ± 1.17	0.34 ± 1.13	t = 1.60	0.112
PTC (°), Mean ± SD	25.61 ± 10.29	25.49 ± 12.15	25.65 ± 9.41	t = −0.12	0.904
LC (°), Mean ± SD	27.73 ± 8.61	26.53 ± 9.91	28.25 ± 7.96	t = −1.41	0.160
T1 tilt (°), Mean ± SD	0.98 ± 6.95	2.04 ± 8.31	0.52 ± 6.24	t = 1.69	0.092
CA (°), Mean ± SD	−0.92± 2.72	−0.96± 2.66	−0.90± 2.75	t =−0.17	0.863
CBD (mm), Mean ± SD	2.72 ± 14.06	2.67 ± 13.49	2.75 ± 14.33	t =−0.05	0.964
TTS (mm), Mean ± SD	11.21 ± 14.73	9.38 ± 14.38	12.00 ± 14.85	t = −1.38	0.170
TK (°), Mean ± SD	21.31 ± 12.35	19.98 ± 11.47	21.89 ± 12.70	t = −1.19	0.234
SVA (mm), Mean ± SD	−4.00 ± 31.58	−3.77 ± 29.27	−4.10 ± 32.60	t = 0.08	0.936
LL (°), Mean ± SD	−37.68 ± 9.72	−38.45 ± 9.34	−37.34 ± 9.88	t = −0.88	0.380
Follow up (months), Mean ± SD	35.84 ± 6.02	35.35 ± 5.95	36.05 ± 6.05	t = −0.89	0.372
UIV-T1(segments), Mean ± SD	1.97 ± 0.96	1.82 ± 0.92	2.03 ± 0.97	t = −1.67	0.097
PTC flexibility (%), M (Q₁, Q₃)	26.09 (14.29, 45.98)	29.17 (16.13, 46.15)	25.00 (14.29, 45.45)	Z = −0.92	0.357
MTC (°), M (Q₁, Q₃)	46.00 (43.00, 56.00)	45.00 (42.00, 51.00)	47.00 (43.00, 57.00)	Z = −1.91	0.056
MTC flexibility (%), M (Q₁, Q₃)	33.33 (22.92, 45.69)	32.50 (22.92, 48.84)	33.33 (22.92, 44.19)	Z = −0.07	0.947
LC flexibility (%), M (Q₁, Q₃)	78.47 (62.50, 90.48)	79.55 (62.31, 90.62)	78.38 (62.50, 90.32)	Z = −0.06	0.953
RSH (mm), M (Q₁, Q₃)	−3.90 (−11.80, 2.10)	−4.00 (−12.00, 2.10)	−3.90 (−11.00, 2.00)	Z = −0.18	0.855
AVT of the MTC (mm), M (Q₁, Q₃)	38.40 (29.70, 47.70)	37.60 (27.60, 44.90)	39.00 (29.80, 49.10)	Z = −1.78	0.075
LIV-CSVL (mm), M (Q₁, Q₃)	2.00 (−8.07, 10.07)	2.00 (−7.40, 10.10)	2.00 (−8.10, 10.00)	Z = −0.48	0.629
CIB, n(%)				χ^2^ = 0.64	0.425
No	224 (79.43)	70 (82.35)	154 (78.17)		
Yes	58 (20.57)	15 (17.65)	43 (21.83)		
Sex, n(%)				χ^2^ = 0.04	0.838
Male	74 (26.24)	23 (27.06)	51 (25.89)		
Female	208 (73.76)	62 (72.94)	146 (74.11)		
Lenke type, n(%)				χ^2^ = 0.15	0.703
1	225 (79.79)	69 (81.18)	156 (79.19)		
2	57 (20.21)	16 (18.82)	41 (20.81)		
Lumbar Modifier, n(%)				χ^2^ = 1.14	0.566
A	208 (73.76)	62 (72.94)	146 (74.11)		
B	59 (20.92)	20 (23.53)	39 (19.80)		
C	15 (5.32)	3 (3.53)	12 (6.09)		
Risser grade, n(%)				-	0.135
0	13 (4.61)	5 (5.88)	8 (4.06)		
1	7 (2.48)	1 (1.18)	6 (3.05)		
2	23 (8.16)	4 (4.71)	19 (9.64)		
3	71 (25.18)	15 (17.65)	56 (28.43)		
4	131 (46.45)	46 (54.12)	85 (43.15)		
5	37 (13.12)	14 (16.47)	23 (11.68)		
TRC, n(%)				χ^2^ = 1.74	0.187
Open	21 (7.45)	9 (10.59)	12 (6.09)		
Closed	261 (92.55)	76 (89.41)	185 (93.91)		

Valid, internal validation set; Train, training set; CB, coronal balance; CIB, coronal imbalance; t, t-test; Z, Mann–Whitney test; χ^2^, Chi-square test; -, Fisher's exact test; SD standard deviation; M, Median; Q₁, first Quartile; Q₃: third Quartile

**Table 2:** Univariate Analysis of Preoperative Morphometric and Surgical Parameters Between the CB and CIB in the Training Set

Variables	Total (*n* = 197)	CB (*n *= 154)	CIB (*n* = 43)	Statistic	*P*
Age (years), Mean ± SD	14.30 ± 1.82	14.34 ± 1.82	14.19 ± 1.85	t = 0.48	0.631
LIV-LSTV (segments), Mean ± SD	0.34 ± 1.13	0.54 ± 1.10	−0.37 ± 0.95	t = 4.95	< 0.001*
PTC (°), Mean ± SD	25.65 ± 9.41	25.49 ± 9.27	26.23 ± 10.00	t = −0.45	0.650
LC (°), Mean ± SD	28.25 ± 7.96	28.63 ± 8.06	26.88 ± 7.53	t = 1.27	0.204
T1 tilt (°), Mean ± SD	0.52 ± 6.24	0.25 ± 6.17	1.50 ± 6.45	t = −1.16	0.247
CA (°), Mean ± SD	−0.90 ± 2.75	−0.86 ± 2.70	−1.04 ± 2.94	t = 0.38	0.704
CBD (mm), Mean ± SD	2.75 ± 14.33	2.71 ± 14.55	2.89 ± 13.64	t = −0.07	0.941
TTS (mm), Mean ± SD	12.00 ± 14.85	12.26 ± 15.29	11.06 ± 13.28	t = 0.47	0.640
TK (°), Mean ± SD	21.89 ± 12.70	21.75 ± 12.23	22.40 ± 14.40	t = −0.30	0.768
SVA (mm), Mean ± SD	−4.10 ± 32.60	−5.79 ± 32.83	1.95 ± 31.38	t = −1.38	0.170
LL (°), Mean ± SD	−37.34 ± 9.88	−37.71 ± 10.19	−36.02 ± 8.64	t = −0.99	0.323
Follow up (months), Mean ± SD	36.05 ± 6.05	36.05 ± 5.89	36.05 ± 6.65	t = 0.01	0.996
UIV-T1(segments), Mean ± SD	2.03 ± 0.97	2.03 ± 0.95	2.05 ± 1.07	t = −0.12	0.903
PTC flexibility (%), M (Q₁, Q₃)	25.00 (14.29, 45.45)	26.14 (14.42, 47.13)	21.43 (12.47, 38.68)	Z = −1.49	0.135
MTC (°), M (Q₁, Q₃)	47.00 (43.00, 57.00)	47.50 (44.00, 57.00)	46.00 (42.00, 55.00)	Z = −1.50	0.133
MTC flexibility (%), M (Q₁, Q₃)	33.33 (22.92, 44.19)	33.87 (21.51, 46.26)	31.67 (24.41, 37.24)	Z = −1.40	0.162
LC flexibility (%), M (Q₁, Q₃)	78.38 (62.50, 90.32)	86.36 (62.50, 91.67)	73.33 (64.58, 77.67)	Z = −3.39	< 0.001*
RSH (mm), M (Q₁, Q₃)	−3.90 (−11.00, 2.00)	−3.65 (−11.65, 2.08)	−5.60 (−11.00, 0.85)	Z = −0.46	0.642
AVT of the MTC (mm), M (Q₁, Q₃)	39.00 (29.80, 49.10)	38.75 (30.02, 48.88)	40.00 (29.25, 49.35)	Z = −0.26	0.791
LIV-CSVL (mm), M (Q₁, Q₃)	2.00 (−8.10, 10.00)	2.00 (−6.83, 10.00)	0.10 (−9.60, 11.60)	Z = −0.51	0.609
Sex, n(%)				χ^2^ = 0.54	0.462
Male	51 (25.89)	38 (24.68)	13 (30.23)		
Female	146 (74.11)	116 (75.32)	30 (69.77)		
Lenke type, n(%)				χ^2^ = 2.96	0.085
1	156 (79.19)	126 (81.82)	30 (69.77)		
2	41 (20.81)	28 (18.18)	13 (30.23)		
Lumbar Modifier, n(%)				χ^2^ = 19.12	< 0.001*
A	146 (74.11)	123 (79.87)	23 (53.49)		
B	39 (19.80)	27 (17.53)	12 (27.91)		
C	12 (6.09)	4 (2.60)	8 (18.60)		
Risser grade, n(%)				-	0.014*
0	8 (4.06)	4 (2.60)	4 (9.30)		
1	6 (3.05)	4 (2.60)	2 (4.65)		
2	19 (9.64)	12 (7.79)	7 (16.28)		
3	56 (28.43)	40 (25.97)	16 (37.21)		
4	85 (43.15)	74 (48.05)	11 (25.58)		
5	23 (11.68)	20 (12.99)	3 (6.98)		
TRC, n(%)				χ^2^ = 0.01	0.931
Open	12 (6.09)	10 (6.49)	2 (4.65)		
Closed	185 (93.91)	144 (93.51)	41 (95.35)		

CB, coronal balance; CIB, coronal imbalance; t: t-test; Z, Mann–Whitney test; χ^2^, Chi-square test; -, Fisher's exact test; SD, standard deviation; M, Median; Q₁: firstQuartile; Q₃: third Quartile; *P < 0.05 indicates a significant difference

### Dimensionality reduction and feature selection

**Fig. 2:**
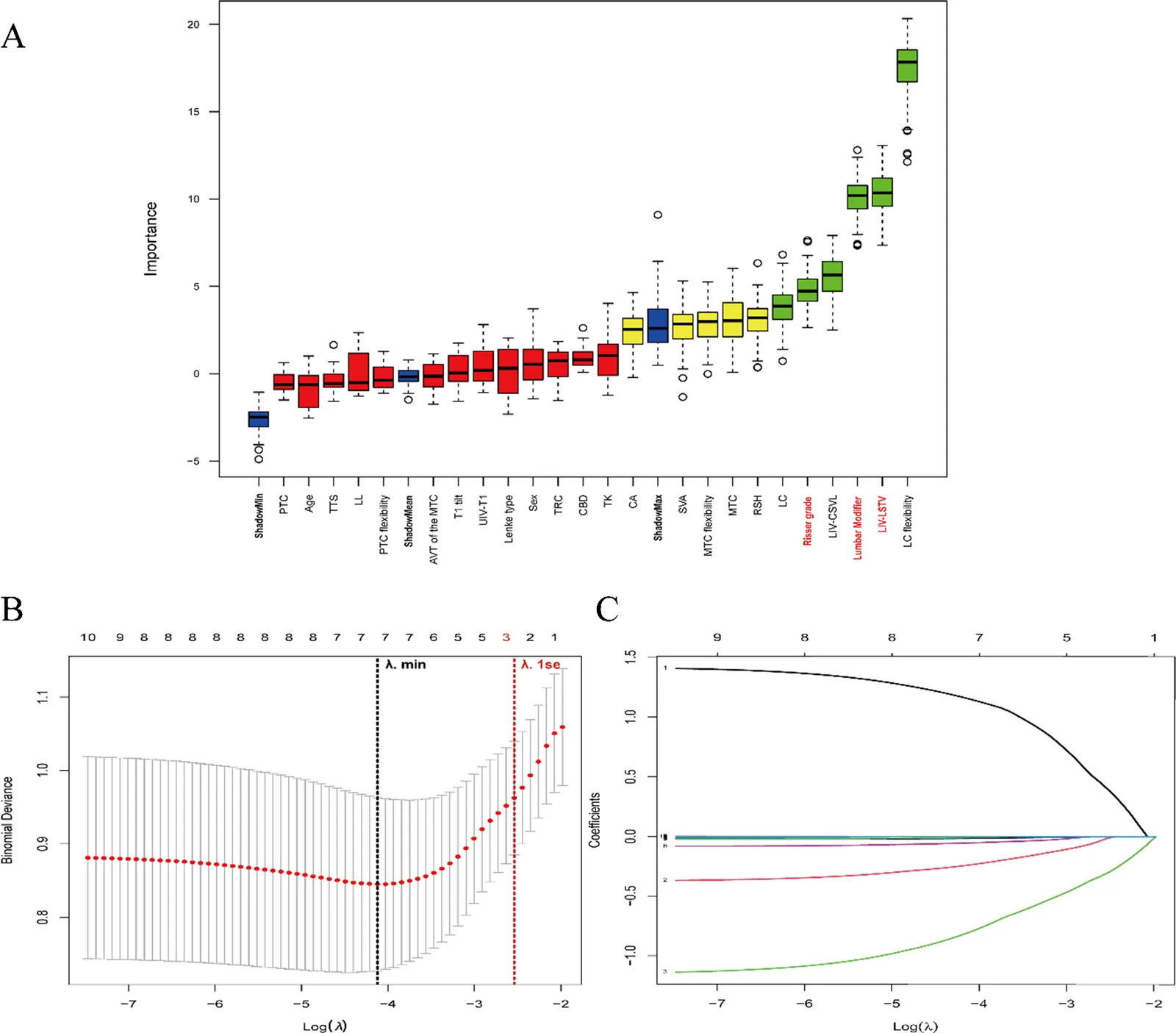
Two-stage dimensionality reduction and feature selection pipeline. (**A**) Boruta algorithm plot ranking the intrinsic importance of candidate variables for initial redundancy elimination. (**B**) LASSO regression plot displaying the partial likelihood (binomial) deviance curve across varying penalty thresholds. (**C**) LASSO coefficient profile plot showing the candidate predictors with vertical dashed lines denoting the optimal tuning parameters (λ_*min*_ and λ_*1se*_). Using the 1-SE criterion, three predictors were retained: LIV-LSTV, Lumbar Modifier, and Risser grade

To isolate robust predictors from the 24 initial candidates, a dual-stage screening pipeline was executed. The Boruta algorithm initially eliminated redundant features, narrowing the pool to ten potential predictors (Fig. 2 A). This subset subsequently underwent LASSO regression with L1 regularization. To prioritize model generalizability and proactively mitigate overfitting, the optimal penalty threshold was defined utilizing the 1-SE criterion (λ_*1se*_ = 0.041, over λ_*min*_ = 0.015) via cross-validation (Fig. 2B,C). Ultimately, this feature-selection process retained the dataset into three predictors: LIV-LSTV, Risser grade, and Lumbar Modifier.

### Model performance evaluation

**Fig. 3:**
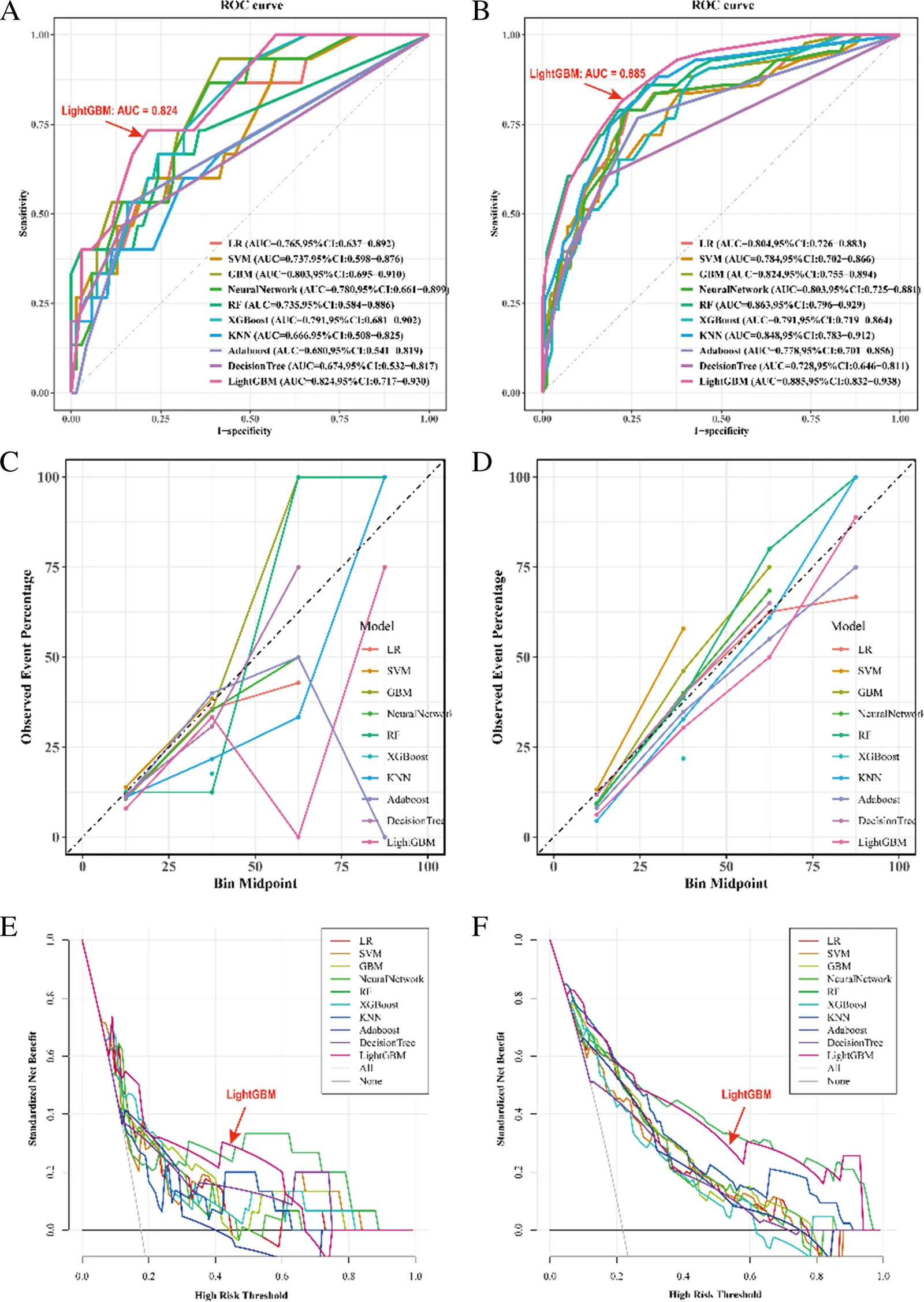
Comprehensive performance evaluation of the ten machine learning architectures. Receiver Operating Characteristic (ROC) curves depicting the predictive discriminative capacity in the internal validation (**A**) and training (**B**) Sets. Calibration plots illustrating diagnostic reliability and probability concordance in the internal validation (**C**) and training (**D**) sets. Decision Curve Analysis (DCA) plots quantifying the standardized net clinical benefit across a continuum of risk thresholds in the internal validation (**E**) and training (**F**) sets. Red arrows highlight the performance of the LightGBM model, including its AUC values in the internal validation (AUC = 0.824) and training (AUC = 0.885) cohorts and its corresponding curves in the DCA plots

With the predictive feature space defined, ten predictive models were comprehensively evaluated (Table 3). Given the potential non-linear biomechanical compensations inherent to AIS, the performance of LightGBM was compared directly with that of the conventional multivariable LR model. Specifically, LightGBM demonstrated the most favourable overall performance within the current dataset, achieving an AUC of 0.885 (95% CI: 0.832–0.938) in the training cohort (Fig. 3B) and 0.824 (95% CI: 0.717–0.930) in the internal validation cohort (Fig. 3A). In the internal validation cohort, LightGBM achieved a numerically higher AUC than multivariable LR (0.824 vs. 0.765) (Fig. 3A), corresponding to an absolute difference of 0.059. LightGBM also showed numerically higher accuracy (0.776 vs. 0.659) and F1 score (0.537 vs. 0.473), together with a lower Brier score (0.118 vs. 0.125) (Table 3). The calibration plots for the internal validation and training cohorts are shown in Fig. 3C and D, respectively. To explore potential net clinical benefit, DCA suggested that LightGBM provided a favorable net benefit across a range of risk thresholds within the current internal validation framework (Fig. 3E,F). These findings should not be interpreted as evidence that use of the model improves surgical decision-making or patient outcomes. Based on its overall discrimination, calibration, and decision-curve performance, LightGBM was selected for subsequent interpretation.

**Table 3:** Predictive Performance and Evaluation Metrics of the Ten Machine Learning Architectures

Data set	Model	Accuracy	Sensitivity	Specificity	Precision	F1 score	Brier score	C index
**Train set**	LR	0.766	0.791	0.760	0.479	0.596	0.130	0.804
	SVM	0.782	0.651	0.818	0.500	0.566	0.155	0.784
	GBM	0.772	0.791	0.766	0.486	0.602	0.136	0.824
	Neural Network	0.772	0.791	0.766	0.486	0.602	0.132	0.803
	RF	0.787	0.791	0.786	0.507	0.618	0.110	0.863
	XGBoost	0.645	0.884	0.578	0.369	0.521	0.164	0.791
	KNN	0.761	0.814	0.747	0.473	0.598	0.118	0.848
	Adaboost	0.741	0.767	0.734	0.446	0.564	0.140	0.778
	Decision Tree	0.777	0.605	0.825	0.491	0.542	0.139	0.728
	**LightGBM**	0.787	0.814	0.779	0.507	**0.625**	**0.104**	**0.885**
**Valid set**	LR	0.659	0.867	0.614	0.325	0.473	0.125	0.765
	SVM	0.753	0.600	0.786	0.375	0.462	0.136	0.737
	GBM	0.647	0.933	0.586	0.326	0.483	0.125	0.803
	Neural Network	0.659	0.867	0.614	0.325	0.473	0.126	0.780
	RF	0.741	0.667	0.757	0.370	0.476	0.122	0.735
	XGBoost	0.741	0.667	0.757	0.370	0.476	0.149	0.791
	KNN	0.671	0.600	0.686	0.290	0.391	0.143	0.666
	Adaboost	0.776	0.533	0.829	0.400	0.457	0.149	0.680
	DecisionTree	0.788	0.467	0.857	0.412	0.437	0.124	0.674
	**LightGBM**	0.776	0.733	0.786	**0.423**	**0.537**	**0.118**	**0.824**

Bold values highlight the selected LightGBM model and its strongest performance metrics. LR denotes a multivariable logistic regression model based on the same three retained predictors as the LightGBM model. LightGBM was selected as the optimal model because it demonstrated the best overall balance of discrimination, calibration, and potential clinical net benefit on decision curve analysis. Valid, internal validation set; Train, training set; LR, Logistic Regression; SVM, Support Vector Machine; GBM, Gradient Boosting Machine; RF, Random Forest; XGBoost, eXtreme Gradient Boosting; KNN, K-Nearest Neighbor

### Algorithmic interpretability and risk deconstruction

**Fig. 4:**
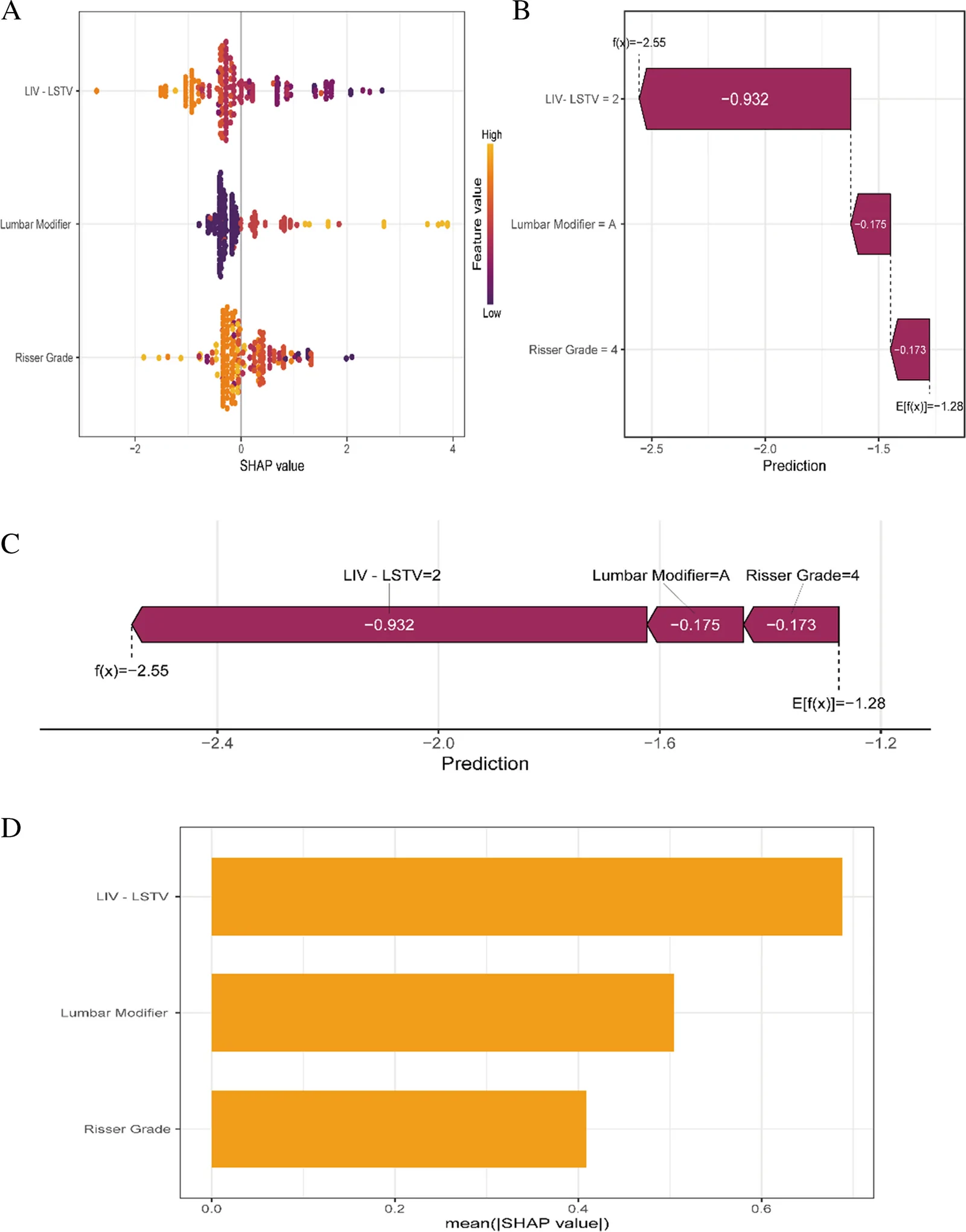
SHapley Additive exPlanations (SHAP) analysis of the optimal LightGBM model. (**A**) SHAP summary (beeswarm) plot illustrating the global impact and distribution of the core predictors. (B) SHAP waterfall plot showing the cumulative directional effect of specific predictors for a representative patient. (**C**) SHAP force plot visualizing the individualized risk prediction driven by distinct variables for a specific patient. (**D**) SHAP variable importance bar plot ranking the overall contribution of individual predictors

To demystify the algorithm's decision-making, SHAP analysis delineated three key predictors contributing to predicted CIB risk: LIV-LSTV, Lumbar Modifier, and Risser grade (Fig. 4A, D). Rather than isolated variables, these features reflected intertwined clinical vulnerabilities. The spatial relationship between the lowest instrumented vertebra and the LSTV was associated with distal biomechanical stability, whereas the Lumbar Modifier and skeletal maturity (Risser grade) captured intrinsic structural adaptability. Beyond global feature importance, waterfall and force diagrams illustrated patient-specific contributions of individual predictors to the model output (Fig. 4B, C). In the representative SHAP example, a LIV-LSTV value of 2, a Lumbar Modifier of A, and a Risser grade of 4 all contributed negatively to the predicted CIB risk, shifting the model output from an expected baseline of − 1.28 to − 2.55.

## Discussion

By analyzing a single-center retrospective dataset and comparing ten distinct machine learning architectures, this study developed an interpretable LightGBM model for postoperative CIB in Lenke 1/2 AIS. Through the SHAP framework, the model identified three key predictors: LIV-LSTV, Lumbar Modifier, and Risser grade. These findings provide a preliminary basis for further evaluation of individualized preoperative risk stratification.

Previous studies have provided an important foundation for identifying factors associated with postoperative coronal alignment. Li et al. highlighted the potential of ML methods to integrate multidimensional data when predicting AIS progression (16) and surgical outcomes. Beauchamp et al. demonstrated the clinical relevance of the touched vertebra criterion for LIV selection in Lenke type 1 and 2 curves (17). Inappropriate LIV selection relative to the compensatory curve has also been linked to postoperative coronal decompensation in patients with Lenke 5C curves (18). The present study extends this work by focusing specifically on postoperative CIB after selective posterior thoracic fusion and by evaluating the combined contributions of LIV-LSTV, Lumbar Modifier, and Risser grade. Unlike traditional linear models, which generally estimate the independent contribution of each variable, LightGBM can capture non-linear relationships and interactions among surgical planning parameters, lumbar curve characteristics, and skeletal maturity. Because the final model was based on only three predictors, a conventional multivariable LR model remains an important comparator. In the internal validation cohort, LightGBM showed a higher AUC and F1 score and a lower Brier score than LR, showing a modest numerical difference in performance. However, these findings do not establish that a more complex ML architecture is universally preferable to a simpler regression-based model. In addition, SHAP analysis provides both global feature importance and patient-specific explanations, enhancing model transparency and clinical interpretability. These findings suggest that postoperative CIB is not attributable to a single anatomical factor but may reflect the interaction between distal instrumentation selection and the compensatory capacity of the unfused lumbar spine. Whether the observed improvement in predictive performance is clinically meaningful requires confirmation in independent external cohorts.

**Fig. 5:**
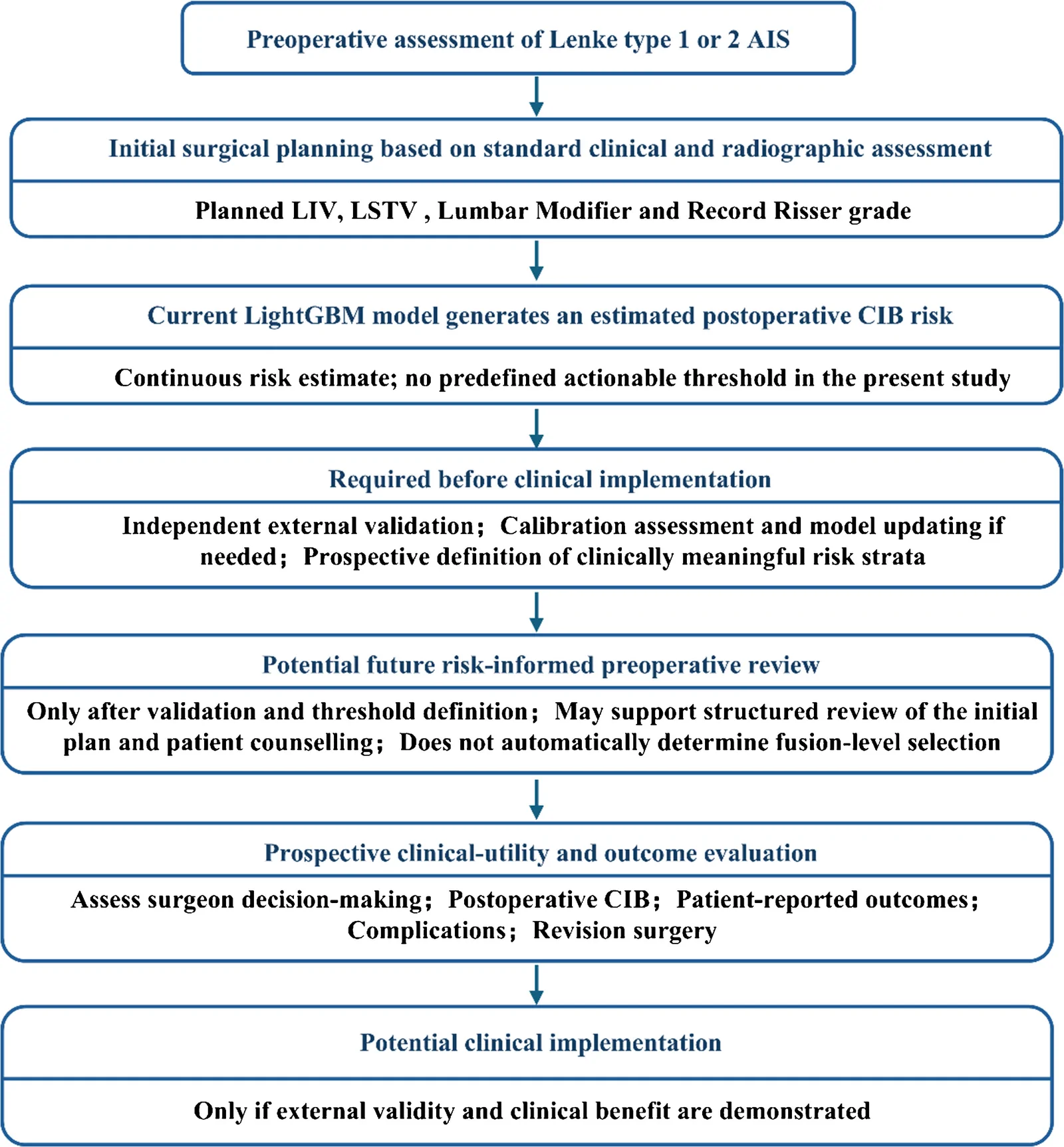
Proposed future validation and implementation pathway for the LightGBM model

From a clinical perspective, if externally validated, the LightGBM model may have potential relevance for future preoperative risk stratification in patients with Lenke 1/2 AIS undergoing selective posterior thoracic fusion. The three predictors required by the model, LIV-LSTV, Lumbar Modifier, and Risser grade, are routinely available during preoperative assessment. After an initial fusion strategy has been proposed, the model could be used to estimate the individualized risk of postoperative CIB. A high predicted risk could prompt further review of the planned LIV, the compensatory capacity of the unfused lumbar curve, and the patient’s skeletal maturity. The predicted risk may also facilitate preoperative counselling by helping patients and their families understand the potential trade-off between preserving lumbar motion segments and reducing the likelihood of postoperative coronal imbalance. Nevertheless, the present study did not evaluate whether use of the model alters surgical decision-making, improves fusion-level selection, reduces postoperative CIB, or improves patient outcomes. Accordingly, the model should be regarded as a preliminary risk-prediction framework requiring independent external validation and prospective clinical-utility evaluation. Figure 5 outlines the proposed future validation and implementation pathway, including multicenter external validation, calibration assessment, risk-threshold definition, clinical-utility evaluation, and outcome assessment before the model can be considered for integration into preoperative planning research.

The definition of postoperative CIB also warrants consideration. In the present study, CIB was defined as a |CBD|> 20 mm at the final follow-up (9). This threshold has been commonly used in previous AIS studies to identify radiographic coronal imbalance. The final follow-up assessment was selected to capture persistent imbalance, as early postoperative coronal decompensation has been reported to improve over time in patients with Lenke 5C curves (19). Persistent coronal imbalance remains clinically relevant because residual coronal decompensation has been associated with poorer patient-reported outcomes, particularly when the imbalance is severe and persistent (20). However, the 20-mm cutoff should be interpreted as a radiographic classification threshold rather than a definitive patient-reported outcome threshold.

Notwithstanding the potential clinical utility of this framework, certain methodological constraints warrant acknowledgment. Most importantly, the model has not undergone external validation. Although the dataset was divided into training and internal validation cohorts, both subsets originated from the same institution and patient population. Therefore, the current split-sample validation assesses internal reproducibility rather than external generalizability. The retrospective, single-centre design of this study inherently limits the external validity and generalizability of the LightGBM model across diverse geographic populations and varying institutional practices. Furthermore, the strict inclusion of selective posterior thoracic fusions limits the immediate extrapolation of these findings to anterior approaches or extensive long-segment instrumentation. The imbalance between the CIB and CB groups (approximately 1:4) represents an additional limitation. While this distribution may reflect the real-world composition of the cohort, the limited number of postoperative CIB events may have affected model stability and predictive performance. Although Boruta and LASSO feature selection, the 1-SE criterion, and cross-validated hyperparameter tuning were used to reduce model complexity and mitigate overfitting, these procedures cannot fully exclude overfitting in the setting of a relatively small number of outcome events. The limited event frequency may also reduce the stability of predictor selection and increase uncertainty in the estimated model performance, particularly in the internal validation cohort. No class-weighting or resampling strategy was applied in the present analysis. Accordingly, the reported performance should be interpreted as preliminary. Concurrently, although the SHAP framework clarifies complex variable associations, extracting definitive biomechanical causality from retrospective data remains intrinsically challenging. A further limitation stems from the model's reliance on static radiographic parameters, which do not capture dynamic equilibrium shifts during gait, load-bearing transitions, or intra-operative correction (21). Future prospective multicenter studies should validate the model in larger cohorts with sufficient numbers of CIB events and assess whether class-weighted algorithms, resampling strategies, and dynamic parameters, such as three-dimensional motion analysis, gait assessment, and intra-operative alignment data, can further refine risk prediction.

## Conclusion

This single-center study developed an interpretable LightGBM model for predicting postoperative CIB in patients with Lenke 1/2 AIS. SHAP analysis identified distal instrumentation selection (LIV-LSTV), lumbar curve morphology (Lumbar Modifier), and skeletal maturity (Risser grade) as the principal predictors. The model demonstrated favorable performance on internal validation using routinely available preoperative variables. If externally validated, it could serve as an adjunct to comprehensive radiographic assessment and surgeon judgement by identifying patients who may warrant closer review of the planned LIV and by supporting counselling regarding the trade-off between coronal stability and preservation of lumbar motion segments. Prospective evaluation in independent multicenter cohorts is required before the model can be used to guide routine clinical decision-making and to establish its generalizability and clinical utility.

## Supplementary information

Below is the link to the electronic supplementary material.

ESM 1 (DOCX 18.0 KB)

## Data Availability

The data analysed during the current study are available from the corresponding author on reasonable request, subject to institutional and ethical restrictions.

## References

[1] Rose PS, Lenke LG (2007) Classification of operative adolescent idiopathic scoliosis: treatment guidelines. Orthop Clin North Am 38(4):521–529. 10.1016/j.ocl.2007.06.001 17945131

[2] Ohrt-Nissen S, Cheung PWH, Kawasaki S, Shigematsu H, Cheung JPY (2024) Curve overcorrection predicts coronal imbalance in selective thoracic fusion in adolescent idiopathic scoliosis. Glob Spine J 14(3):856–861. 10.1177/21925682221124526 PMC11192118 36045481

[3] Fang Y, Li J, Hu Z, Zhu Z, Qiu Y, Liu Z (2024) Postoperative coronal imbalance in Lenke 5C adolescent idiopathic scoliosis: evolution, risk factors, and clinical implications. Neurospine 21(3):903–912. 10.14245/ns.2448544.272 PMC11456952 39363470

[4] Majdouline Y, Aubin CE, Robitaille M, Sarwark JF, Labelle H (2007) Scoliosis correction objectives in adolescent idiopathic scoliosis. J Pediatr Orthop 27(7):775–781. 10.1097/BPO.0b013e31815588d8 17878784

[5] Lenke LG, Edwards CC, Bridwell KH (2003) The Lenke classification of adolescent idiopathic scoliosis: how it organizes curve patterns as a template to perform selective fusions of the spine. Spine (Phila Pa 1976) 28(20):S199-207. 10.1097/01.BRS.0000092216.16155.33 14560193

[6] Ohrt-Nissen S, Luk KDK, Samartzis D, Cheung JPY (2020) Selection of the lowest instrumented vertebra in main thoracic adolescent idiopathic scoliosis: is it safe to fuse shorter than the last touched vertebra? Eur Spine J 29(8):2018–2024. 10.1007/s00586-020-06398-4 32242322

[7] Qiu XS, Wang ZW, Qiu Y et al (2013) Preoperative pelvic axial rotation: a possible predictor for postoperative coronal decompensation in thoracolumbar/lumbar adolescent idiopathic scoliosis. Eur Spine J 22(6):1264–1272. 10.1007/s00586-013-2695-7 PMC3676562 23392555

[8] Anari JB, Tatad A, Cahill PJ, Flynn JM, Harms Study Group (2020) The impact of posterior spinal fusion (PSF) on coronal balance in adolescent idiopathic scoliosis (AIS): a new classification and trends in the postoperative period. J Pediatr Orthop 40(9):e788–e793. 10.1097/BPO.0000000000001622 32658159

[9] Jiang H, Shao W, Xu E et al (2018) Coronal Imbalance after Selective Posterior Thoracic Fusion in Patients with Lenke 1 and 2 Adolescent Idiopathic Scoliosis. Biomed Res Int 2018:3476425. 10.1155/2018/3476425 PMC6304582 30627550

[10] Mannem A, Cheung PWH, Kawasaki S, Shigematsu H, Cheung JPY (2021) What determines immediate postoperative coronal balance and delayed global coronal balance after anterior spinal fusion for Lenke 5C curves? Eur Spine J 30(7):2007–2019. 10.1007/s00586-021-06807-2 33721065

[11] Gavotto A, Risser A, Séverac F, Charles YP (2024) Influence of age and severity of Lenke 5 or 6 idiopathic scoliosis on postoperative quality of life in adult patients. Orthop Traumatol Surg Res 110(7):103742. 10.1016/j.otsr.2023.103742 37913868

[12] Banno T, Yamato Y, Oba H et al (2022) Risk factors and clinical impact of persistent coronal imbalance after posterior spinal fusion in thoracolumbar/lumbar idiopathic scoliosis. J Neurosurg Spine 37(6):883–892. 10.3171/2022.5.SPINE22385 35901689

[13] Bao H, Yan P, Qiu Y, Liu Z, Zhu F (2016) Coronal imbalance in degenerative lumbar scoliosis: prevalence and influence on surgical decision-making for spinal osteotomy. Bone Joint J 98-B(9):1227–1233. 10.1302/0301-620X.98B9.37273 27587525

[14] Bellini V, Valente M, Bertorelli G et al (2022) Machine learning in perioperative medicine: a systematic review. J Anesth Analg Crit Care 2(1):2. 10.1186/s44158-022-00033-y PMC8761048 37386544

[15] Lundberg SM, Erion G, Chen H et al (2020) From local explanations to global understanding with explainable AI for trees. Nat Mach Intell 2(1):56–67. 10.1038/s42256-019-0138-9 PMC7326367 32607472

[16] Li L, Wong MS (2024) The application of machine learning methods for predicting the progression of adolescent idiopathic scoliosis: a systematic review. BioMed Eng OnLine 23(1):80. 10.1186/s12938-024-01272-6 PMC11308564 39118179

[17] Beauchamp EC, Lenke LG, Cerpa M et al (2020) Selecting the “touched vertebra” as the lowest instrumented vertebra in patients with Lenke type-1 and 2 curves: radiographic results after a minimum 5-year follow-up. J Bone Joint Surg Am 102(22):1966–1973. 10.2106/JBJS.19.01485 32804885

[18] Hu B, Yang X, Yang H et al (2018) Coronal imbalance in Lenke 5C adolescent idiopathic scoliosis regarding selecting the lowest instrumented vertebra: lower end vertebra versus lower end vertebra +1 in posterior fusion. World Neurosurg 117:e522–e529. 10.1016/j.wneu.2018.06.070 29929029

[19] Matsumura A, Iwamae M, Namikawa T et al (2021) Spontaneous improvement of postoperative coronal imbalance following selective thoracolumbar-lumbar fusion in Lenke 5C adolescent idiopathic scoliosis. World Neurosurg 151:e241–e249. 10.1016/j.wneu.2021.04.024 33866027

[20] Anari JB, LaValva SM, Flynn JM, Tatad AM, the Harms Study Group (2020) Severe persistent coronal imbalance following instrumented posterior spinal fusion for adolescent idiopathic scoliosis. Spine Deform 8(6):1295–1304. 10.1007/s43390-020-00153-x 32500442

[21] Paradkar R, Regan C, Bernhardt K, Kaufman KR, Milbrandt TA, Larson AN (2025) Reactive balance in adolescent idiopathic scoliosis: a prospective motion analysis study. J Clin Med 14(5):1715. 10.3390/jcm14051715 PMC11900989 40095864

