# Supplementary material for: Predicting postoperative coronal imbalance in Lenke 1/2 adolescent idiopathic scoliosis: A machine learning model with clinical interpretability
Source: Int Orthop. 2026 Jul 18;50(11):2963–75. doi: 10.1007/s00264-026-06953-6 (PMC13694364; doi:10.1007/s00264-026-06953-6)
Supplement: Supplementary file 1 [file 264_2026_6953_MOESM1_ESM.docx]

**Supplementary material 1. Optimal Hyperparameters Configurations for the Evaluated Machine Learning Architectures.**

| **Model** | **Parameters** |
| --- | --- |
| SVM | sigma = 0.001, C = 0.5 |
| GBM | n.trees = 100, interaction.depth = 3, shrinkage = 0.01, n.minobsinnode = 5 |
| Neural Network | size = 3, decay = 0.6 |
| RF | mtry = 2 |
| XGBoost | nrounds = 10, max_depth = 4, eta = 0.1, gamma = 0.5, colsample_bytree = 0.5, min_child_weight = 1, subsample = 0.6 |
| KNN | kmax = 12, distance = 1 |
| Adaboost | mfinal = 2, maxdepth = 4 |
| Decision Tree | cp = 0.007751938 |
| LightGBM | min_data=1, learning_rate =1, num_threads=2, verbosity=1, num_iterations=5, early_stopping_round=3 |

Note: LR : Logistic Regression, SVM: Support Vector Machine, GBM: Gradient Boosting Machine, RF: Random Forest, XGBoost: eXtreme Gradient Boosting, KNN: K-Nearest Neighbor.
